# Sustainable plant-based diets promote rainbow trout gut microbiota richness and do not alter resistance to bacterial infection

**DOI:** 10.1186/s42523-021-00107-2

**Published:** 2021-07-05

**Authors:** David Pérez-Pascual, Ana Elena Pérez-Cobas, Dimitri Rigaudeau, Tatiana Rochat, Jean-François Bernardet, Sandrine Skiba-Cassy, Yann Marchand, Eric Duchaud, Jean-Marc Ghigo

**Affiliations:** 1 Unité de Génétique des Biofilms, Institut Pasteur, UMR CNRS 2001, 75015 Paris, France; 2 Biologie des Bactéries Intracellulaires Institut Pasteur, UMR CNRS 3525, 75015, Paris, France; 3 Unité Infectiologie Expérimentale Rongeurs et Poissons, INRAE, Université Paris-Saclay, 78350 Jouy-en-Josas, France; 4 Université Paris-Saclay, INRAE, UVSQ, VIM, 78350 Jouy-en-Josas, France; 5 INRAE, Univ Pau & Pays Adour, E2S UPPA, NUMEA, 64310 Saint-Pée-sur-Nivelle, France; 6 Le Gouessant, F-22402 Lamballe, France

**Keywords:** Rainbow trout, Gut microbiota, Sustainable aquaculture diet, *Flavobacterium psychrophilum*

## Abstract

**Background:**

Farmed fish food with reduced fish-derived products are gaining growing interest due to the ecological impact of fish-derived protein utilization and the necessity to increase aquaculture sustainability. Although different terrestrial plant proteins could replace fishmeal proteins, their use is associated with adverse effects. Here, we investigated how diets composed of terrestrial vegetal sources supplemented with proteins originating from insect, yeast or terrestrial animal by-products affect rainbow trout (*Onchorynchus mykiss*) gut microbiota composition, growth performance and resistance to bacterial infection by the fish pathogen *Flavobacterium psychrophilum* responsible for frequent outbreaks in aquaculture settings.

**Results:**

We showed that the tested regimes significantly increased gut bacterial richness compared to full vegetal or commercial-like diets, and that vegetal diet supplemented with insect and yeast proteins improves growth performance compared to full vegetal diet without altering rainbow trout susceptibility to *F. psychrophilum* infection.

**Conclusion:**

Our results demonstrate that the use of insect and yeast protein complements to vegetal fish feeds maintain microbiota functions, growth performance and fish health, therefore identifying promising alternative diets to improve aquaculture’s sustainability.

**Supplementary Information:**

The online version contains supplementary material available at 10.1186/s42523-021-00107-2.

## Background

Aquaculture is a fast-growing animal-food production sector that currently supplies over 50% of the fish and seafood for human consumption [1]. Fish farming still relies on marine fish-derived fishmeal as the primary protein source in fishfood [2]. Considering the decline of available marine natural resources, the replacement of fishmeal by proteins originating from terrestrial plant sources is actively investigated [2–4]. However, although more sustainable than fishmeal, the use of full plant-based diets in aquaculture is associated with reduced growth yield, modification of fish metabolism and increased sensitivity to diseases potentially due to unbalanced amino acid profile, presence of anti-nutritional factors and alteration of fish microbiota [5–7].

Alternatives to terrestrial plant protein sources are currently sought to replace fish-derived proteins, including extracts from insects, yeast, and terrestrial animal by-products [8–10]. These protein sources display interesting nutritional value and could mitigate the adverse effects of plant-derived proteins [11, 12]*.* However, the consequences on fish growth of introducing new ingredients in fish feeds need to be carefully evaluated as fish are sensitive to dietary changes [13] and the stability of gut microbial communities is an essential factor affecting fish health [14, 15]. Dietary changes can impact growth performance, digestibility as well as host immunity and resistance to diseases [12, 16–18]. The protection provided by the microbiota against pathogens is indeed of particular importance in high-density aquaculture settings, where farmed fish are often plagued by disease outbreaks that threaten the economic sustainability of the farming industry [19]. Among the most prominent fish pathogens, *Flavobacterium psychrophilum* is a common freshwater bacterium and the causative agent of Bacterial Cold-Water Disease and Rainbow Trout Fry Syndrome. This pathogen affects a wide range of temperate and cold-water fish worldwide, especially salmonids such as rainbow trout (*Onchorynchus mykiss*), one of the leading aquaculture species [20].

Here, we investigated the short-term consequences of protein source substitution on gut microbiota composition, growth performance and resistance to *F. psychrophilum* infection in rainbow trout. We compared diets exclusively composed of terrestrial vegetal sources to diets supplemented with proteins originating from insect, yeast or terrestrial animal by-products. We showed that insects and yeast constitute promising protein complements to vegetal fish feeds by increasing gut bacterial richness and improving growth performance compared to a vegetal diet or a commercial-like diet, without altering fish resistance to an experimental *F. psychrophilum* infection. These properties support the potential interest of these ingredients in maintaining growth performance and microbiota functions while contributing to the aquaculture’s sustainable development.

## Results

### Changes of protein sources in diet affect the α-diversity of rainbow trout gut microbiota

**Fig. 1:**
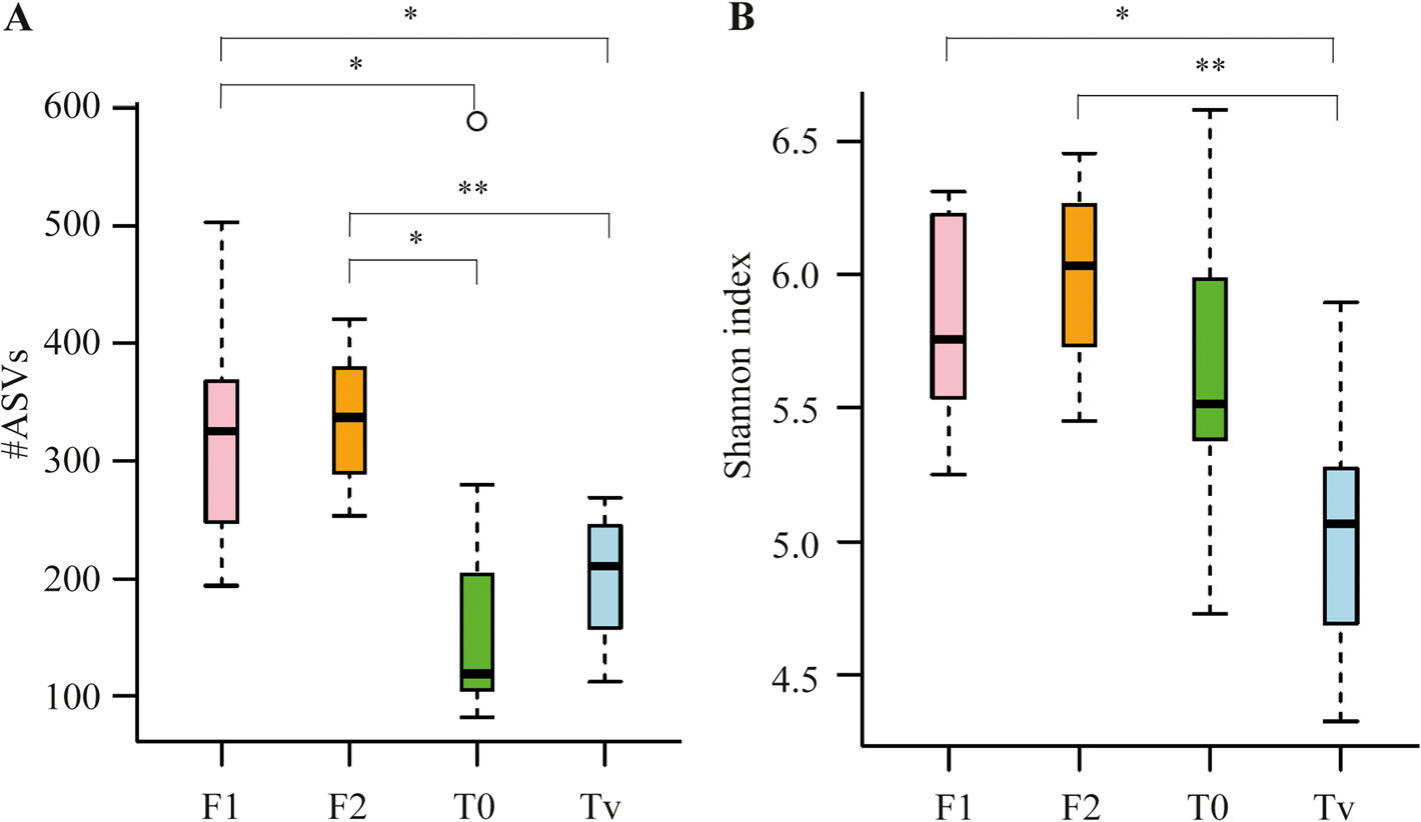
Bacterial α-diversity metrics (**A**: Amplicon Single Variants (ASV) richness, and **B**: Shannon index) of the gut of rainbow trout submitted to four different diets (*n* = 9 fish/diet). T_0_: commercial fishmeal-rich diet; Tv: full terrestrial plant-based diet devoid of fishmeal; F1: plant-based diet in which 11% of plant proteins have been replaced by 5.5% of proteins from yeast (YEA) and 5.5% of proteins from insect larvae (INS); F2: plant-based diet in which 16.5% of plant proteins have been replaced by 5.5% YEA, 5.5% INS and 5.5% processed animal proteins (PAP). The kruskal-wallis pairwise test was used to perform the statistical comparisons among diets. Significance: *, *p*-value < 0.05; **, *p*-value < 0.01; ***, *p*-value < 0.001

To evaluate the effect of diet modification on microbiome composition, we performed a 16S rRNA sequencing analysis of the microbial gut content of rainbow trout subjected to four different diets: T_0_, a commercial fishmeal-rich diet; Tv, a full terrestrial plant-based diet devoid of fishmeal; F1, a plant-based diet in which 11% of plant proteins have been replaced by 5.5% of proteins from yeast (YEA) and 5.5% of proteins from insect larvae (INS); and F2, a plant-based diet in which 16.5% of vegetal proteins have been replaced by 5.5% YEA, 5.5% INS and 5.5% processed animal proteins (PAP) (Supplementary Table S1). An average of 340,612 reads for the amplified 16S rRNA gene was obtained per sample. After quality filtering, denoising, and chimeric removal, we obtained an average of 237,752 good-quality reads (Supplementary Table S2). The diversity analyses were performed according to the sample’s depth with the lowest number of reads (43,800). The intestinal microbiome of fish fed the experimental diets F1 and F2 had a significantly higher richness than those fed the control Tv and T_0_ diets (F1 vs T_0_ and Tv, *p*-values = 0.03; F2 vs T_0_ and Tv; *p*-values = 0.02 and 0.002, respectively), with about 350 Amplicon Single Variants (ASVs) detected in the F1 and F2 groups and a lower number of ASVs (about 200) in the Tv and T_0_ control groups (Fig. 1A). No differences were found in the number of ASVs among the control diets Tv and T_0_ (*p*-value > 0.05), although more diversity was found in the T_0_ group reaching the limit of significance (*p*-value = 0.05). The Shannon index was used to examine diversity distribution and showed that microbiota from fish fed the experimental diets F1 and F2 were significantly more diverse (*p* < 0.01) than that of fish fed the full-vegetal Tv control diet (Fig. 1B). The results showed that intestinal microbiota diversity of rainbow trout was higher when fish were fed the diets containing alternative protein sources.

### The origin of diet protein sources shapes the composition of rainbow trout gut microbiota

**Fig. 2:**
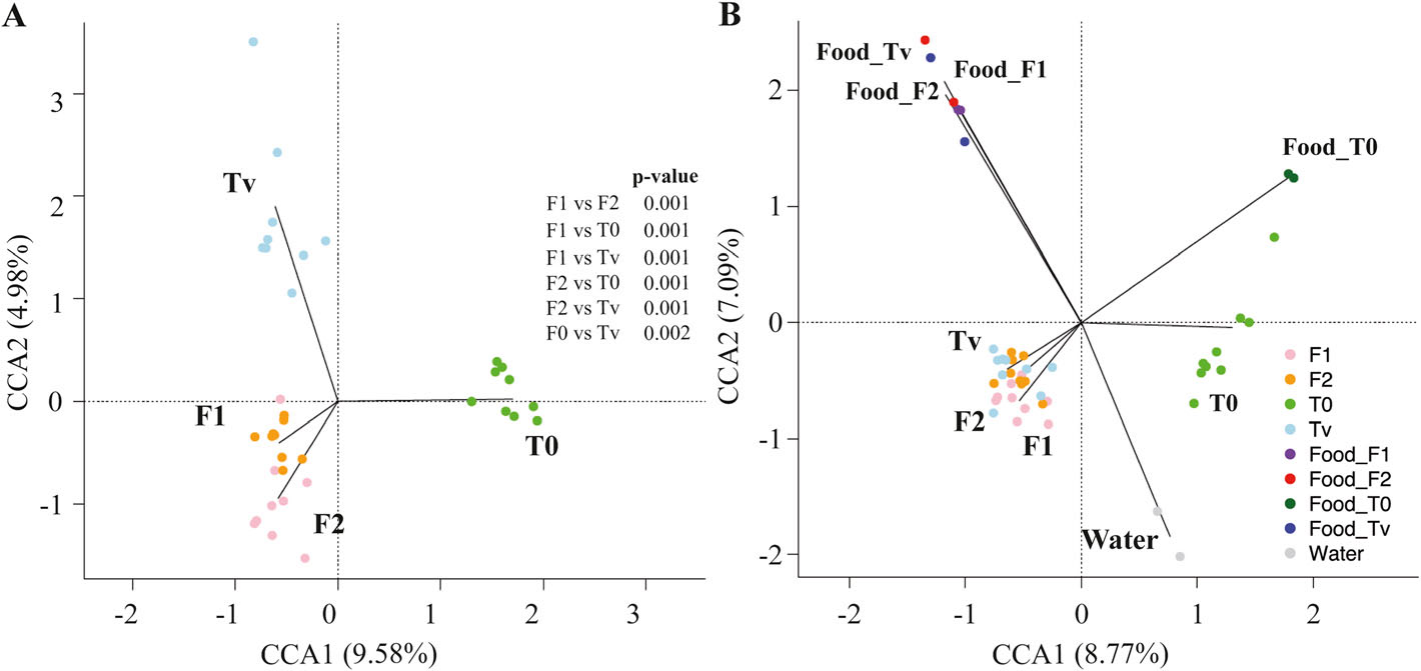
Canonical Correspondence Analysis (CCA) for **A**, gut microbial composition comparisons between rainbow trout fed four different dietary protein sources for three months (*n* = 9 fish/diet); and **B**, sample types including microbial structure associated to each food and raising water. Each dot represents an individual sample plot according to its microbial profile at the ASV level. Results of Permutational multivariate analysis of variance (PERMANOVA) coupled with the Bray-Curtis dissimilarity matrix are reported. Significance was set at *p* < 0.05. T_0_: commercial fishmeal-rich diet; Tv: full terrestrial plant-based diet devoid of fishmeal; F1: plant-based diet in which 11% of plant proteins have been replaced by 5.5% of proteins from yeast (YEA) and 5.5% of proteins from insect larvae (INS); F2: plant-based diet in which 16.5% of plant proteins have been replaced by 5.5% YEA, 5.5% INS and 5.5% processed animal proteins (PAP)

To determine whether the different experimental diets could alter the overall composition of rainbow trout gut bacterial communities, we compared samples from different groups (β-diversity). The microbial community variations among groups were explored by Canonical Correspondence Analysis (CCA) applied to the Bray-Curtis dissimilarity matrix. Fishmeal replacement by terrestrial vegetal proteins clearly affects the gut microbial structure since samples from T_0_ and Tv clustered in two separate groups. The microbiota under diets T_0_ and Tv shows a higher variability compared to F1 and F2, which profiles were more homogeneous. Furthermore, the inclusion of new protein sources such as insect and yeast proteins in F1 and the same sources supplemented with PAP in F2 resulted in distinct bacterial communities among the groups compared to the control diets (Fig. 2A). The significance of the observed clusters was confirmed by the PERMANOVA test coupled to the Bray-Curtis dissimilarity matrix (*p* < 0.001) (Fig. 2A, Supplementary Table S3). Moreover, the weighted UniFrac analysis (phylogenetic distance) also revealed that community phylogenetic diversity and relative abundance of ASVs were significantly different (*p* < 0.001) among groups, confirming what was found with the ecological distance Bray-Curtis dissimilarity (Supplementary Table S3). Interestingly, when we compared the microbiota of water, feed, and fish intestine, we observed that rainbow trout gut microbial composition is more similar to that of feed than water, the latter being significantly different from the fish gut composition, independently of the diet (Fig. 2B). Altogether, these results showed that while diet modification has a significant impact, the microbiota present in surrounding water has little effect on rainbow trout’s bacterial community structure.

### Comparison of rainbow trout microbiota composition among experimental groups reveals a diet-independent core microbiota

**Fig. 3:**
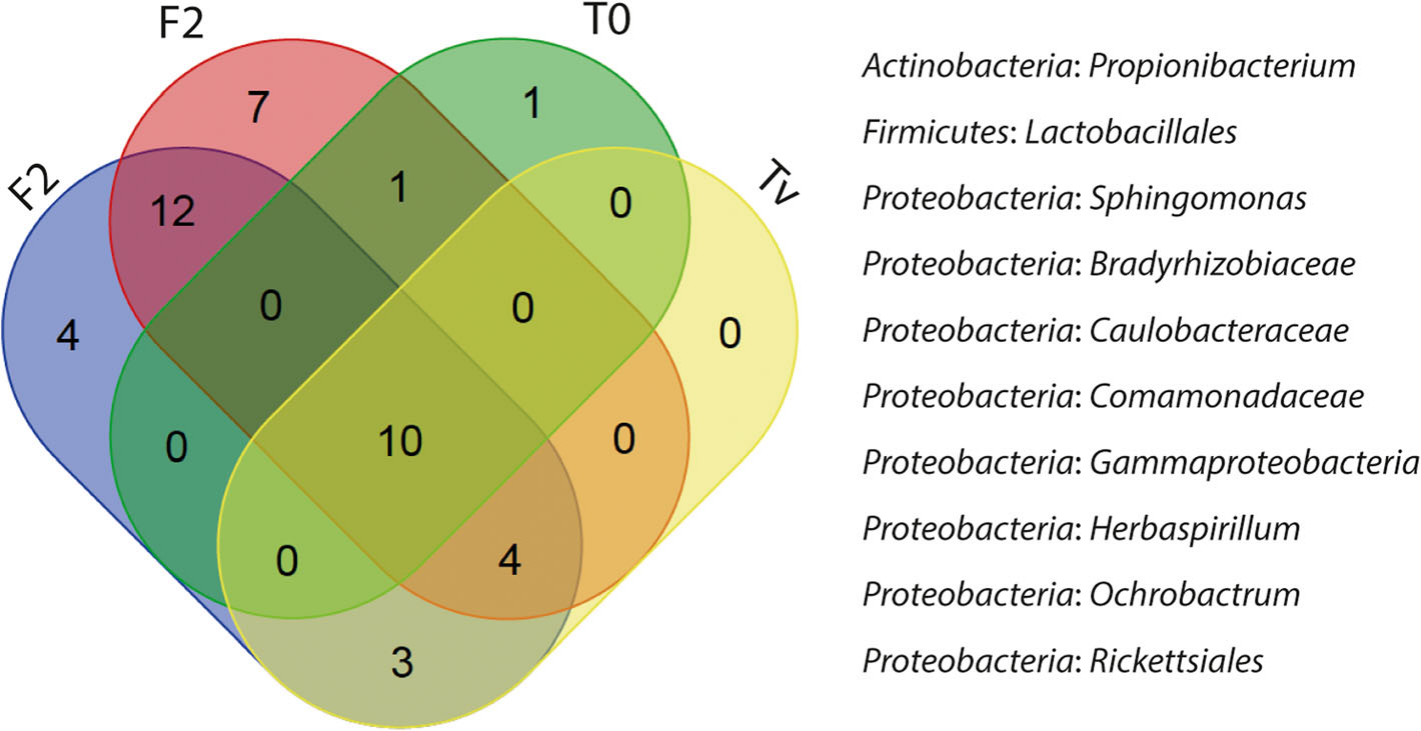
Venn diagram representing unique and shared Amplicon Single Variants (ASVs) in the gut of rainbow trout fed four different diets (*n* = 9 fish/diet). The core microbiome was defined as the ASVs present in all samples, regardless of diet. Only taxa presented in all samples of each group were included

In rainbow trout, as in many other fish species, a set of bacteria is present in the gastrointestinal tract irrespective of the diet, forming its “core microbiota” [21], which is hypothesized to contribute to the beneficial relationship between host and microbiota [22]. We determined that the intestinal core microbiota across all individuals tested was composed of ten shared ASVs, including eight *Proteobacteria* (orders *Sphingomonadales*, *Hyphomicrobiales*, *Caulobacterales*, *Burkholderiales*, *Rickettsiales* and class *Gammaproteobacteria*), one *Firmicutes* (order *Lactobacillales*), and one *Actinobacteria* (order *Propionibacteriales*) (Fig. 3). A total of 12 ASVs were specific of diet, while the two diets sharing more taxa were F1 and F2, with 12 ASVs in common. Some dominant and previously reported members of rainbow trout microbiota, including *Lactobacillales*, *Comamonadaceae*, *Rickettsiales,* and *Bradyrhizobiaceae* were included in this core composition [21, 23–25]. Some ASVs could not be taxonomically assigned to the genus level, suggesting that (1) the rainbow trout microbial diversity is far from being fully described; (2) the selected 16S rRNA region cannot discriminate between close genera; or (3) the taxa are not found due to gaps in the database. These results indicated that a core microbiota is stably associated with rainbow trout gut, regardless of the used diets.

### Distinct dietary protein sources alter rainbow trout gut bacterial community

**Fig. 4:**
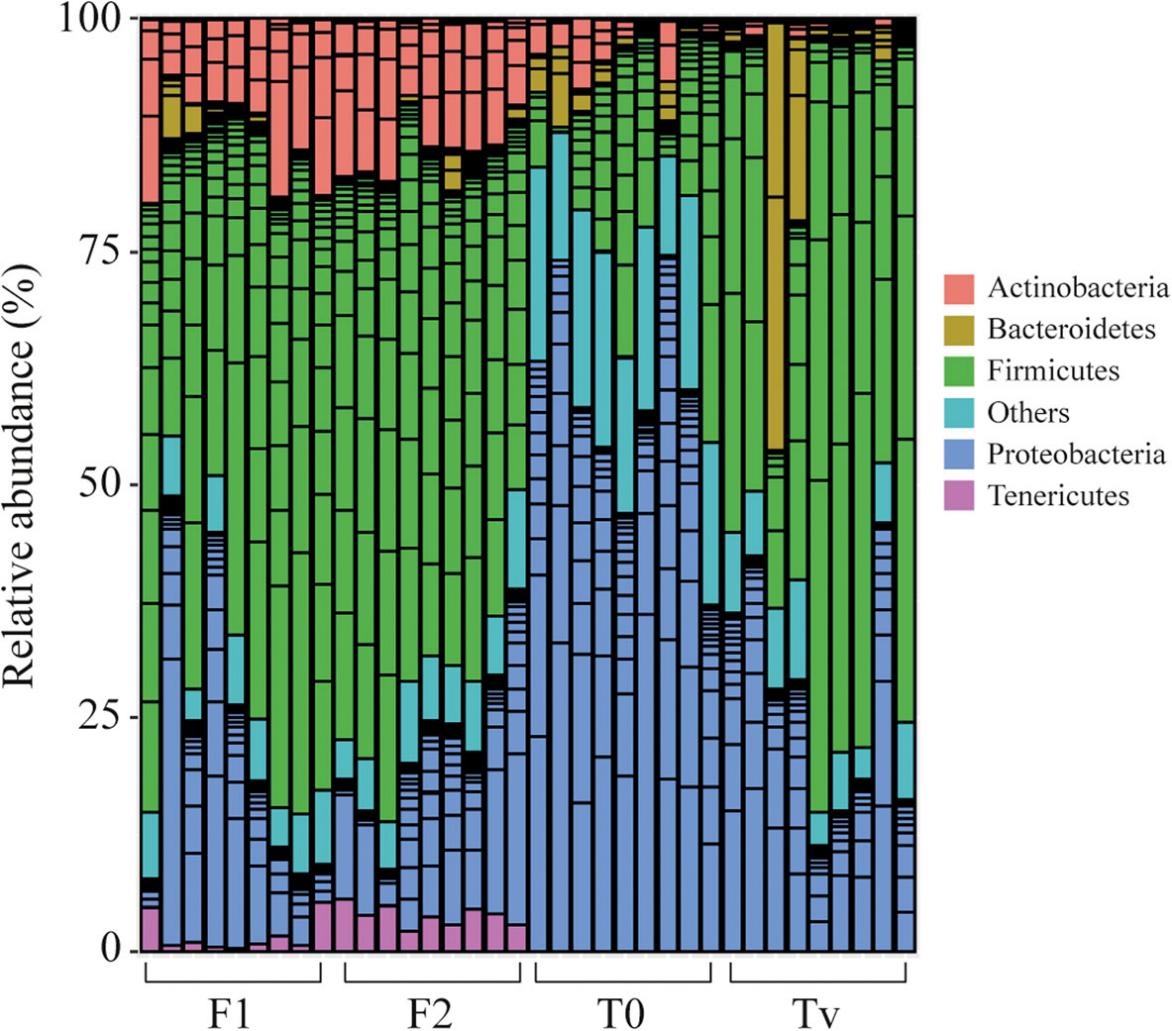
Relative abundance (%) of the overall most prevalent bacterial phyla of the gut of rainbow trout fed different diets

To determine whether the different experimental diets can alter gut microbiota composition, an overall taxonomic characterization of the bacterial community was conducted. A total of 20 different bacterial phyla were identified among which *Firmicutes*, *Proteobacteria*, *Actinobacteria*, and *Bacteroidetes* were dominant, regardless of the diet used. However, fish fed the control vegetal diet (Tv) showed an increase in the relative abundance of the phylum *Firmicutes* (58.89%) as well as a decrease in *Proteobacteria* (29.83%) compared to fish fed the T_0_ diet, rich in fishmeal (23.08 and 64.61%, respectively) (Wilcoxon signed-rank test: *Proteobacteria p*-value = 0.0001645; *Firmicutes p*-value = 0.002756) (Fig. 4). Further, fish fed the experimental diets F1 and F2 showed a significant increase in the abundance of *Firmicutes* (*p*-values = 0.0002879, 8.227e^− 05^) and *Tenericutes* (*p*-values = 0.000161, 0.000161) and a significant decrease of *Proteobacteria* (*p*-values = 0.0001645, 4.114e^-05^), compared to the control commercial (T_0_) group. When fish fed both F1 and F2 experimental diets were compared to those fed the Tv full-vegetal diet, an increase of *Actinobacteria* was detected (*p*-values = 4.114e^− 05^, 4.114e^− 05^) (Fig. 4, Supplementary Figure S1).

To account for differences in sequencing depth, we conducted a differential abundance analysis of the detected microbiota on a proportional abundance table agglomerated at the species level using Analysis of Composition of Microbiomes with Bias Correction (ANCOM-BC) [26]. ANCOM-BC analysis revealed that five species belonging to the order *Lactobacillales* were significantly more abundant in the Tv group compared to fish fed the T_0_ fishmeal-rich diet (*p* < 0.05, ANCOM-BC) (Table 1). These ASVs consist of two species of *Lactobacillus* genus (*Lactobacillus hamsteri* and *L. paraplantarum*), two members of the family *Leuconostocaceae*, and one identified as *Streptococcus agalactiae* (Table 1).

**Table 1:** Mean differences of taxa absolute abundance between groups (natural log scale) and standard errors (SEs). The analysis is based on the “Analysis of Compositions of Microbiomes with Bias Correction” (ANCOM-BC). Only taxa with significantly different abundance between diets are shown (adjusted *p*-value < 0.05, Bonferroni correction)

Phylum	Class	Order	Family	Genus	Species	Mean difference	SE	q-value
**Tv vs T**_**0**_								
*Firmicutes*	*Bacilli*	*Lactobacillales*	*Lactobacillaceae*	*Lactobacillus*	*hamsteri*	5.71	1.24	0.0002
*Firmicutes*	*Bacilli*	*Lactobacillales*	*Leuconostocaceae*	*Lactobacillus*	*paraplantarum*	5.49	1.24	0.0005
*Firmicutes*	*Bacilli*	*Lactobacillales*	*Streptococcaceae*	*Streptococcus*	*agalactiae*	4.69	1.25	0.0104
*Firmicutes*	*Bacilli*	*Lactobacillales*	*Leuconostocaceae*			5.01	0.94	4.78e-06
*Firmicutes*	*Bacilli*	*Lactobacillales*	*Leuconostocaceae*	*Pediococcus*		6.09	1.27	8.94e-05
**Tv vs F1**								
*Firmicutes*	*Bacilli*	*Lactobacillales*	*Lactobacillaceae*	*Lactobacillus*		−3.78	0.94	0.0036
*Firmicutes*	*Bacilli*	*Bacillales*	*Bacillaceae*			−3.06	0.76	0.0032
*Firmicutes*	*Bacilli*	*Lactobacillales*	*Leuconostocaceae*	*Weissella*	*hellenica*	−7.49	1.02	1.53e-11
*Actinobacteria*	*Actinobacteria*	*Actinomycetales*	*Corynebacteriaceae*	*Corynebacterium*	*aurimucosum*	−5.76	0.95	6.15e-07
*Actinobacteria*	*Actinobacteria*	*Actinomycetales*				−3.90	0.60	6.33e-09
**Tv vs F2**								
*Firmicutes*	*Bacilli*	*Bacillales*	*Bacillaceae*			−3.14	0.74	0.0013
*Firmicutes*	*Bacilli*	*Lactobacillales*	*Lactobacillaceae*	*Lactobacillus*		−3.65	0.94	0.0057
*Firmicutes*	*Bacilli*	*Lactobacillales*				−1.95	0.51	0.0068
*Actinobacteria*	*Actinobacteria*	*Actinomycetales*	*Corynebacteriaceae*	*Corynebacterium*	*aurimucosum*	−5.72	0.99	6.15e-07
*Actinobacteria*	*Actinobacteria*	*Actinomycetales*	*Corynebacteriaceae*	*Corynebacterium*		−5.32	0.78	6.21e-10
*Actinobacteria*	*Actinobacteria*	*Actinomycetales*				−3.95	0.65	8.02e-08
**F1 vs F2**								
*Firmicutes*	*Bacilli*	*Lactobacillales*	*Leuconostocaceae*	*Weissella*	*hellenica*	−5.72	0.83	5.74e-10
*Actinobacteria*	*Actinobacteria*	*Actinomycetales*	*Streptomycetaceae*			0.95	0.27	0.0382
*Tenericutes*	*Mollicutes*	RsaHF231				1.48	0.36	0.0030
*Firmicutes*	*Bacilli*	*Lactobacillales*				1.47	0.30	0.0001

While conducting differential abundance analysis between the experimental diets F1 and F2 and the Tv vegetal control diet, ANCOM-BC detected a significant increase of 5 and 6 taxa, in groups F1 and F2, respectively (Table 1). Four of those taxa with increased abundance are shared in both experimental diets, i.e. 2 *Firmicutes* (one identified at the genus level as *Lactobacillus*, and the other at the family level as *Bacillaceae*), and 2 *Actinobacteria* (both belonging to the genus *Corynebacterium*). These results suggest that the increase of these taxonomic groups is mainly associated with the ingredients included in the experimental diet F1 (i.e., yeast and insect proteins), while the PAP included in diet F2 are not involved in the increase. Furthermore, fish fed F1 also showed an increase of another species identified as *Weisella hellenica* (family *Lactobacillaceae*) (Table 1). In the group fed diet F2, the abundance of an additional species belonging to the phylum *Actinobacteria* was higher than in those fed the Tv vegetal control diet (Table 1).

When the taxonomic composition of the microbiota of fish fed diets F1 and F2 are compared, the higher abundance of *W. hellenica* in F1 fed fish is confirmed. Interestingly, the F2 group displayed an increase of three different taxa compared to F1 fed fish: one species belonging to the phylum *Firmicutes* (order *Lactobacillales*), one *Tenericutes* (class *Mollicutes*), and one *Actinobacteria* (family *Streptomycetaceae*).

We tested the association of diet-related variables (dry material, proteins (%), lipids (%), energy, and cinders) with the gut microbiome composition (Supplementary Table S4). The protein (%) was the only variable that showed a significant association with the gut composition (aGLMM-MiRKAT, *p*-value = 0.00019996).

### The use of alternative protein sources in diets impacts rainbow trout feed efficiency and growth performance

**Fig. 5:**
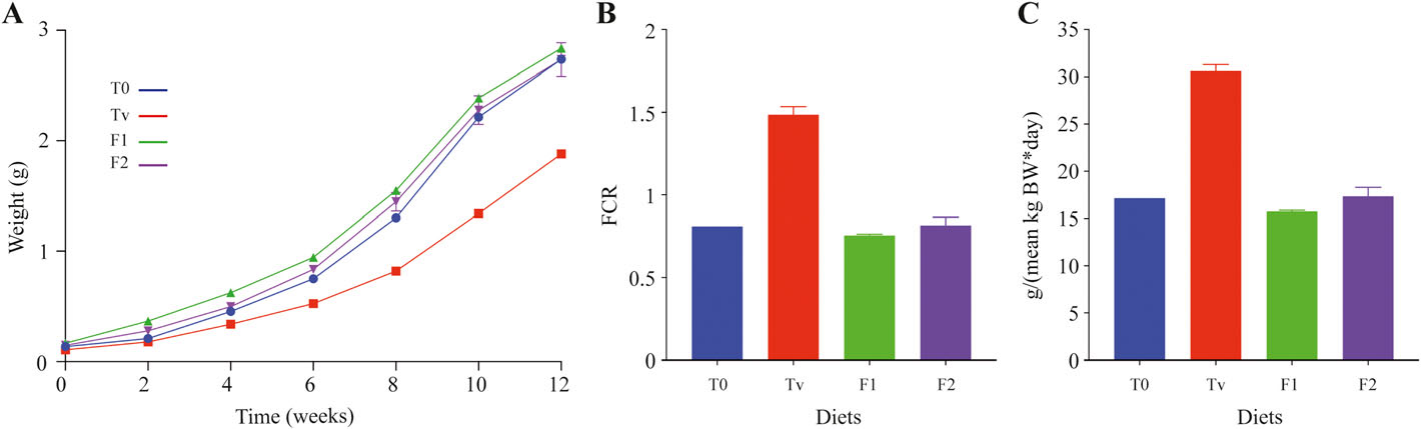
Body weight gain, Feed Conversion Ratio (FCR) and daily feed intake. **A** Evolution of body weight gain of rainbow trout fed with different experimental diets from the first feeding and during 12 weeks**.** Mean body weights for each diet are presented as mean ± standard error. **B** Feed Conversion Ratio (FCR) were calculated per tank as follows: FCR = feed intake (g)/ fish weight gain (g) during the 12 weeks. **C** Daily feed intake was calculated for each tank as follows: feed consumed (g) / mean kg body weight.day. Mean values for each diet are presented as mean ± standard error. T_0_: commercial fishmeal-rich diet (*n* = 1); Tv: full terrestrial plant-based diet devoid of fishmeal (*n* = 2); F1: plant-based diet in which 11% of plant proteins have been replaced by 5.5% of proteins from yeast (YEA) and 5.5% of proteins from insect larvae (INS) (*n* = 2); F2: plant-based diet in which 16.5% of plant proteins have been replaced by 5.5% YEA, 5.5% INS and 5.5% processed animal proteins (PAP) (*n* = 2)

We tested how plant-based feeds supplemented by either insect, yeast or processed animal proteins affected rainbow trout growth performance compared to conventional fishmeal-based diets. For the entire feeding trial duration, observed mortality was 7, 10.5, 5, and 12.5% in fish fed T_0_, Tv, F1, and F2, respectively. Fish fed both experimental diets F1 or F2 displayed a similar final body weight (2.84 ± 0.02 and 2.73 ± 0.10 g) (*P* > 0.05) (Fig. 5A). When groups fed the experimental F1 and F2 diets were compared to those fed the terrestrial-plant based diet Tv (1.88 ± 0.01 g), a significantly higher final body weight was found in the two former groups (*P* < 0.05) (Fig. 5A). Interestingly, no significant difference was observed between the final body weights of fish fed both experimental diets, F1 and F2, and the fishmeal-based diet (2.74 ± 0.01 g) (*P* > 0.05) (Fig. 5A).

The tested diets exhibited similar Feed Conversion Ratio (FCR) except for the control full-vegetal Tv diet, for which the FCR value (Fig. 5B) almost doubled. A similar pattern was observed for the daily feed intake, which increased for fish fed the Tv diet (Fig. 5C). Altogether these results highlight a strong degradation of the feed efficiency in fish fed the Tv diet that was not mitigated by an enhanced feed intake, therefore resulting in lower growth performances. However, these results also showed that supplementation of an entirely plant-based diet with proteins originating from insects, terrestrial animal by-products, and/or yeast restored the fish growth performance at equivalent levels compared to fishmeal-based diet.

### Changes of protein sources in fish diet do not increase rainbow trout susceptibility to an experimental *Flavobacterium psychrophilum* infection

**Fig. 6:**
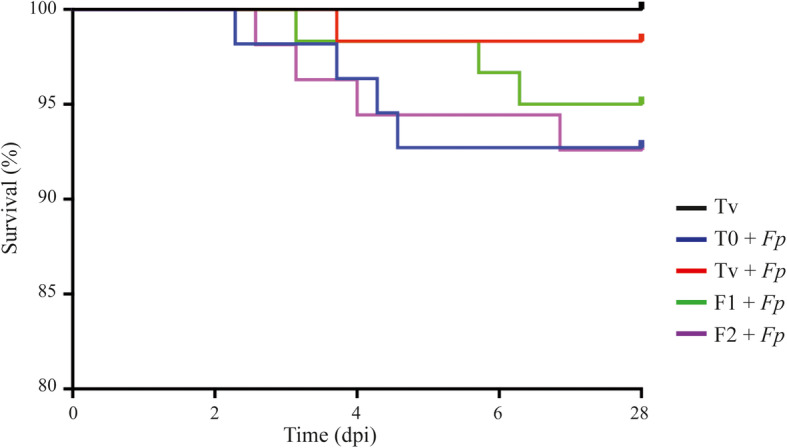
Kaplan–Meier survival curves of rainbow trout following infection by immersion challenge with *F. psychrophilum* strain FRGDSA 1882/11. Fish fed the Tv, T_0_, F1, and F2 diets were infected for 24 h with 2 × 10^7^ CFU mL^−1^ in a final volume of 10 L. The results correspond to the survival percentage during 28 days post-infection (dpi) of two replicates (*n* = 60 fish per condition). Kaplan-Meier survival data was analyzed by log-rank (Mantel-Cox) test

Diet modification and potential changes in the commensal microbial composition can influence the rainbow trout susceptibility to pathogens [27]. *F. psychrophilum* commonly infects the skin and gills of fish, and can adhere to and damage the intestinal epithelium [28, 29]. To determine whether the observed changes in the gut microbial community of rainbow trout can modify its susceptibility to bacterial infection following the feeding period, we exposed 60 fish (duplicates of 30 fish) of each different dietary group to *F. psychrophilum* using an immersion challenge model that supposedly mimics the natural infection route better than the usual injection route [30]. After fish exposition to the virulent *F. psychrophilum* strain FRGDSA 1882/11 for 24 h, no significant difference in fish mortality was observed between those fed the T_0_ commercial and the Tv vegetal based control diets (13.5 vs 1.5%, respectively) (Fig. 6). Interestingly, the F1 and F2 experimental diets did not significantly affect the susceptibility of fish to infection compared to the Tv control diet, though an upward trend could indeed be observed (5 vs 16.5% mortality, respectively) (Fig. 6). None of the non-challenged control fish died during the experiment. *F. psychrophilum* was re-isolated from the spleen of dead or moribund fish from all infected groups. These results showed that the diet modifications tested did not affect rainbow trout susceptibility to *F. psychrophilum* infection.

## Discussion

In sustainable aquaculture, aquafeeds using terrestrial plant-based proteins are gaining increasing importance to cope with the limited availability of fishmeal and reduce the ecological impact of fishmeal utilization. Reliance on fishmeal drastically increases the environmental footprint of farmed fish, because fishmeal-based feeds require harvest from wild fish stocks, further damaging marine ecosystems [3, 31]. However, the use of alternative plant protein sources can induce nutritional imbalance and low palatability in some carnivorous fish species. Together with the presence of anti-nutritional factors, the use of terrestrial plant-based proteins in fish feeds could damage the intestinal tract, reduce nutrient absorption, and fish growth [3]. Whereas previous studies analyzed the effect of fishmeal partial or total replacement by single alternative protein sources on fish growth performance and microbiota composition [8, 32–36], our study assessed the impact of a full replacement of fishmeal with a mix of proteins from different sources. We confirmed that a full terrestrial plant ingredients feed (Tv) resulted in a significantly reduced growth in rainbow trout [37–39]. In contrast, we showed that supplementing a full vegetal diet with proteins from insect and yeast (F1 diet) restored growth performances similar to those obtained in fish fed a fishmeal-rich diet (T_0_). This is consistent with recent studies demonstrating an increase of final body weight and improved feed efficiency of adult rainbow trout fed a plant-based diet supplemented with either insect protein-extract or yeast proteins [32, 33, 40].

Fish gut microbial communities are highly dynamic and respond rapidly to variations in selective local pressures such as diet modification [15, 41]. According to our findings, the gut bacterial community of rainbow trout fry is dominated by members of the phyla *Firmicutes*, *Proteobacteria, Actinobacteria,* and *Bacteroidetes* in the four dietary groups. These phyla usually represent up to 90% of fish intestinal microbiota in different marine and freshwater species [12, 13]. In rainbow trout, as in many other fish species, a set of bacteria occurs in the gastrointestinal tract irrespective of the diet, forming its “core microbiota” [21]. In our study, we identified 10 such core bacterial ASVs, some of which had already been identified in the core microbiota of rainbow trout gut [21, 23–25]. While no significant difference in α-diversity was observed between gut microbiota of fish fed the T_0_ fishmeal-rich diet and the Tv full-terrestrial plant-based diet, our results showed that both experimental diets F1 and F2 significantly increased gut bacterial richness compared to the Tv and T_0_ control diets. This increase could be explained by the introduction of proteins from insect larvae in fish diet, as previously reported in rainbow trout [23, 42]. A higher bacterial diversity could be beneficial since reduced diversity leads to a low colonization resistance capacity, allowing opportunistic pathogens to easily colonize the gastrointestinal tract of fish [43]. Conversely, the additional inclusion of 5.5% proteins from terrestrial animal by-products did not affect intestinal α-diversity, as shown by the lack of significant differences between F1 and F2. Similarly, replacing fishmeal with a mix of terrestrial animal by-product meals and plant proteins did not induce significant changes in gut microbial richness and α-diversity in rainbow trout [8].

Interestingly, ß-diversity analyses revealed a significant relationship between diet type and fish intestine microbiota, as shown by the clustering of samples by diet. Full replacement of fishmeal by terrestrial-plant proteins induces an increase of the relative abundance of the phylum *Firmicutes*, while *Proteobacteria* decreased, as previously reported in rainbow trout [8, 39, 44, 45]. ANCOM-BC analysis showed that the terrestrial plant-based Tv diet induced a significant increase of five species belonging to the order *Lactobacillales* compared to fish fed the T_0_ fishmeal-rich diet. Thus, the full replacement of fishmeal by terrestrial plants derived proteins in T_v_ diet induced an increase in Lactic Acid Bacteria (LAB) such as *Lactobacillus*, *Pediococcus* and *Streptococcus*. These results are in line with previous studies that also showed that the inclusion of terrestrial plant proteins in rainbow trout diet induces a significant increase of several LAB among other microorganisms [21, 39, 44, 45]. Interestingly, several strains belonging to the genus *Pediococcus* displayed probiotic properties in vitro [46] and in vivo in rainbow trout [47]. In other fish species, feeds containing plant derivate indigestible fiber and fermentable polysaccharides were associated with an increase of LAB that can utilize such substrates for their metabolism and growth [48–50]. LAB are considered the most promising bacteria to be used as probiotics in aquaculture due to their ability to stimulate the host’s gastrointestinal development, digestive function, mucosal tolerance, immune response, and to improve disease resistance. However, in addition to the numerous beneficial LAB, several important fish-pathogenic bacteria also belong to this group [51]. This is the case of *S. agalactiae*, which relative abundance is increased in the T_v_ full-terrestrial plant-based diet. *S. agalactiae* has been reported to affect different wild and farmed fish species, particularly the Nile tilapia (*Oreochromis niloticus*) [52].

Samples from fish fed the experimental diets F1 and F2 both significantly clustered separately from those fed the T_0_ and Tv control diets. Similar to full-terrestrial plant-based fed fish, a significant increase in the relative abundance of the phylum *Firmicutes*, and a decrease of *Proteobacteria* were observed in fish fed both experimental diets compared to the T_0_ group. In addition, the supplementation with alternative proteins sources induced a significant increase of the phyla *Tenericutes* and *Actinobacteria* compared to both the T_0_ and Tv diets. Several members of the phyla *Tenericutes* and *Firmicutes* might contribute to the digestion of complex polysaccharides, as suggested in rainbow trout [53] and other fish species [54]. The increase of the phylum *Actinobacteria* may be due to the introduction of chitin-rich insect hydrolyzed proteins [23, 55], since members of this phylum, such as the genus *Streptomyces,* are particularly efficient decomposers of chitinous material [56]. The increase of *Actinobacteria* could be explained by the increased abundance of two species belonging to this phylum, including one identified as *Corynebacterium aurimucosum*, that have been detected as more abundant by ANCOM-BC analysis in the gut microbiote of fish fed the F1 and F2 experimental diets.

The effect on the phylum *Firmicutes* can also be observed at the ASV level, where the F1 and F2 diets induced a significant increase of members of the orders *Lactobacillales* and *Bacillales* in fish gut microbiota. The introduction of poultry by-product proteins (PAP) in the F2 diet slightly altered the microbial profile observed in F1-fed fish by increasing three different species belonging to the phyla *Actinobacteria* (family *Streptomycetaceae*), *Firmicutes* (order *Lactobacillales)* and phylum *Tenericutes* (class *Mollicutes*). Also, the relative abundance of *W. hellenica* is significantly decreased in F2 group compared to the F1 group. This fact may be of importance since *W. hellenica* is a LAB species that have been used as a probiotic in fish, due to its antimicrobial activity against several fish pathogens [57].

Based on observed changes in fish gut microbiota composition induced by diet manipulation, we tested the influence of these modifications on the rainbow trout susceptibility to the notorious salmonid pathogen *F. psychrophilum.* Strikingly, the new feed formulations did not affect the susceptibility of rainbow trout to *F. psychrophilum* infection compared to fish fed a fishmeal-rich diet. This promising result may be linked to the fact that the enrichment in bacterial diversity induced by the alternative protein sources increased the resistance of the host to infections. In addition, the ß-glucans in insect and yeast derived ingredients were shown to successfully stimulate the immune response of fish and their resistance to pathogens [58, 59].

## Conclusions

In conclusion, compared to a commercial-like fishmeal-based diet, supplementation with a mix of alternative proteins from insect and yeast reduced the adverse effects of plant-based diet on growth performance and increased the richness of the gut microbiota without impacting the resistance to *F. psychrophilum*. These results open new perspectives to enhance the efficiency of fish feeds devoid of fishmeal and contribute to the development of a more sustainable aquaculture.

## Methods

### Fish and breeding conditions

The four diets were formulated by Le Gouessant Aquaculture® company and produced in small scale by INRAE facilities (Supplementary Table S1 and S4). The T_0_ control diet was a commercial-like diet containing fishmeal and fish oil. The three other diets (Tv, F1 and F2) were composed of a blend of plant proteins and oil sources and microalgae as DHA source. In diets F1 and F2, 11 and 16.5% of the plant ingredients were replaced by alternative protein sources, respectively. In diet F1, 11% of plant proteins were replaced by 5.5% of yeast protein fraction from *Saccharomyces cerevisiae* (Nutrisaf® 503, Phileo by Lesaffre, France) and 5.5% of hydrolyzed insect larvae meal (*Hermetia illucens*) (Copalis, France). In diet F2, an additional replacement of 5.5% of plant proteins by the same amount of processed animal proteins (composed of a blend of poultry blood meal and by-product meal) was carried out. Pellets were obtained using an extrusion cooking process (CLEXTRAL BC45 extruder with double screw, Firminy, France) under a 44.5 bar pressure and a 52 °C mean temperature, then dried at 40 °C with an air flux for a minimum of 2 h.

The rainbow trout standard line (SY*AUT) selected by INRAE-PEIMA was used. Eyed eggs (210 degrees days) entered the facilities after disinfection (Romeiode®) and were incubated at 10 °C in a recycling aquaculture system (RAS) into dechlorinated water. Before first feeding, fry were randomly distributed in 30-l aquariums (200 fish/aquarium); 2 aquariums were used for each experimental diet (Tv, F1 and F2) and the control diet (T_0_). Fish were reared for 14 weeks (90 days) from the first feeding with ad libitum feed rations (4–5 meals/day). During this growth phase, feed rations and fish were weighed every 15 days to evaluate the zootechnical performance (growth and FCR). FCR was calculated as follows: FCR = feed intake (g)/ fish weight gain (g). At the end of the growth period, some of the fish were sampled for microbial analysis, while others were transferred to the “pathogen zone” of the experimental facilities (NSB2) for experimental infection trials.

### Fish infection challenges

Infection challenges were performed by immersion as previously described [30]. Fish (3 g) were transferred to continuous-flow aquaria for infection experiments. Groups of 30 fish fed the control and experimental diets were challenged in duplicates (*n* = 60 per diet). The highly virulent *F. psychrophilum* strain FRGDSA 1882/11 [60] was grown in TYES broth (0.4% (w/v) tryptone, 0.04% (w/v) yeast extract, 0.05% (w/v) MgSO_4_ 7H_2_O, 0.02% (w/v) CaCl_2_ 2H_2_O, 0.05% (w/v) D-glucose, pH 7.2) at 200 rpm and 18 °C to late-exponential phase (OD_600_ of 1). Bacterial cultures were directly diluted (100-fold) into aquaria containing 5 l of water and the water flow was stopped for 8 h. Then, the volume of aquarium water was increased to 10 l and fish were maintained in contact with bacteria for an additional 16 h to reach a total duration of 24 h. Planktonic bacteria were subsequently removed by restoring the flow. Instead of a bacterial culture, sterile TYES broth was used for the control group. Bacterial counts were determined at the beginning and at the end of the immersion challenge by plating serial dilutions of water samples on TYES agar. Water was maintained at 10 °C under continuous aeration during the experiment. Virulence was evaluated according to fish mortality during 28 days postinfection. Kaplan-Meier survival data were analyzed using the log-rank (Mantel-Cox) test with GraphPad Prism 8.4.0 (GraphPad Software, San Diego, CA, USA).

### Sampling of intestinal contents

Sampling of intestinal content to characterize gut bacterial communities was performed at the end of the trial on day 90. Nine fish per diet were randomly chosen and euthanized by an overdose of filtered tricaine methane sulfonate solution (Sigma, 300 mg L^− 1^). The whole intestine (proximal and distal parts) was dissected under sterile conditions and the intestinal content was squeezed out individually into an Eppendorf tube and kept at − 80 °C until DNA extraction [61]. A total of 36 intestinal content samples were collected. In addition, four 2-ml samples of input water as well as 250 mg samples of each experimental feed were collected for analysis of their bacterial content.

### DNA extraction and 16S rRNA gene sequencing

Total bacterial DNA was extracted from each gut content sample using the QIAmp DNA Microbiome kit (Qiagen), following the manufacturer’s instructions. DNA concentrations were measured using a Nanodrop™ 1000 (Thermo Scientific Ltd.). Samples with DNA concentration > 10 ng μl^− 1^ with an absorbance ratio A260:A280 of > 1.8 were kept for further analysis. 16S rRNA gene amplification, library construction and sample sequencing were performed by PCR of the V3-V4 region with 5 ng of DNA and 200 nM of specific primers: forward primer 5′ TCGTCGGCAGCGTCAGATGTGTATAAGAGACAGCCTACGGGNGGCWGCAG-3′, and reverse primer 5′-GTCTCGTGGGCTCGGAGATGTGTATAAGAGACAGGACTACHVGGGTATCTAATCC-3′ [62]. PCR products were purified using solid-phase reversible immobilization (SPRI) paramagnetic bead-based technology (AMPure XP beads, Beckman Coulter) with a bead:DNA ratio of 0.7:1 (v/v) and quantified using a Qubit® 2.0 Fluorometer (Invitrogen). Sequencing was performed at a final concentration of 4 pM with a 10% PhiX control library spike-in on a MiSeq instrument (Illumina Inc.) with 600 cycles v3 chemistry to generate 2 × 300 bp paired-end reads.

### Sequence data processing and statistics

Raw 16S rRNA data were processed with QIIME2 (version qiime2–2019.4) [63]. The quality filtering, primer sequences removal, denoising, and Amplicon Single Variants (ASVs) estimation were performed using the DADA2 algorithm [64] implemented in the QIIME2 pipeline.

To estimate α- and β-diversity metrics on the ASVs data, we used the “core-metrics-phylogenetic” function from QIIME2. Analyses were performed at a depth of 43,800 reads per sample. We focused on the Shannon index (diversity) and the number of observed ASVs (richness) from the α-diversity metrics. A Kruskal-Wallis-Pairwise test with a Benjamini & Hochberg correction was applied to compare the metrics between groups of samples statistically. The metrics were represented in boxplots in R [65].

Differences in composition (β-diversity) between the different types of diet or sample were explored using a Canonical Correspondence Analysis (CCA) using the Bray-Curtis dissimilarity matrix. In addition, a PERMANOVA test was performed on Bray-Curtis dissimilarity and Weighted Unifrac matrixes to determine whether the differences in bacterial composition between specific groups were significant. The CCA and the PERMANOVA analyses applied were implemented in the R package “Vegan” [66, 67].

The microbiome R package was used to determine the core by using the “core_members” function. Only taxa presented in all the samples of each group were included. The Venn diagram was drawn using an online tool: http://bioinformatics.psb.ugent.be/webtools/Venn/. A bar plot of the bacterial composition was plotted by using the R package phyloseq [68].

Bacterial composition comparisons between diet conditions were performed by using Analysis of Composition of Microbiomes with Bias Correction (ANCOM-BC) [26]. This method identifies those taxa with statistically significant different abundances between groups of samples.

The association between the microbial community composition and quantitative variables was performed using the aGLMM-MiRKAT analysis implemented in the R package “GLMM-MiRKAT” [69]. The GLMM-MiRKAT is a distance-based kernel association test based on the generalized linear mixed model (GLMM) correcting type I error rates.

## Supplementary Information

**Additional file 1: Supplementary Figure S1.** Relative abundance (%) of the overall most prevalent bacteria at genus (or the next lowest taxonomic level) of the gut of rainbow trout fed different diets, food and raising water samples. **Supplementary Table S1.** Feed formulations for rainbow trout (g/100 g feed) of commercial-like feed (T_0_), full terrestrial-vegetal feed (Tv) and experimental feeds (F1 and F2). **Supplementary Table S2.** Summary of the sequencing pre-processing. The number and percentage of reads that passed the quality filter, denoising, chimera checking, and the paired-end merged reads are shown. **Supplementary Table S3.** PERMANOVA test comparison of bacterial composition under experimental diets. The distance matrix was based on the Bray-Curtis dissimilarity matrix or Weighted-Unifrac distance. Q-values under 0.05 were considered significant. **Supplementary Table S4.** Crude protein, lipid, fiber, chitin, starch and cinder percentage of each feed formulations for rainbow trout (% of dry matter) of commercial-like feed (T_0_), full terrestrial-vegetal feed (Tv) and experimental feeds (F1 and F2).

## Data Availability

Raw sequences were deposited in the European Nucleotide Archive (ENA) under accession number PRJEB41459.

## Abbreviations

16S rRNA: 16S ribosomal ribonucleic acid
YEA: Yeast
INS: Insect
PAP: Processed animal proteins
ASV: Amplicon Single Variants
CCA: Canonical Correspondence Analysis
ANCOM: Analysis of composition of microbiomes
