# Supplementary material for: Sustainable plant-based diets promote rainbow trout gut microbiota richness and do not alter resistance to bacterial infection
Source: Anim Microbiome. 2021 Jul 5;3:47. doi: 10.1186/s42523-021-00107-2 (PMC8256591; doi:10.1186/s42523-021-00107-2)
Supplement: Supplementary file 1 — Additional file 1: Supplementary Figure S1. Relative abundance (%) of the overall most prevalent bacteria at genus (or the next lowest taxonomic level) of the gut of rainbow trout fed different diets, food and raising water samples. Supplementary Table S1. Feed formulations for rainbow trout (g/100 g feed) of commercial-like feed (T0), full terrestrial-vegetal feed (Tv) and experimental feeds (F1 and F2). Supplementary Table S2. Summary of the sequencing pre-processing. The number and percentage of reads that passed the quality filter, denoising, chimera checking, and the paired-end merged reads are shown. Supplementary Table S3. PERMANOVA test comparison of bacterial composition under experimental diets. The distance matrix was based on the Bray-Curtis dissimilarity matrix or Weighted-Unifrac distance. Q-values under 0.05 were considered significant. Supplementary Table S4. Crude protein, lipid, fiber, chitin, starch and cinder percentage of each feed formulations for rainbow trout (% of dry matter) of commercial-like feed (T0), full terrestrial-vegetal feed (Tv) and experimental feeds (F1 and F2). [file 42523_2021_107_MOESM1_ESM.docx]

**SUPPLEMENTARY MATERIALS**

**SUPPLEMENTARY FIGURES**

**Supplementary Figure S1:** Relative abundance (%) of the overall most prevalent bacteria at genus (or the next lowest taxonomic level) of the gut of rainbow trout fed different diets, food and raising water samples.


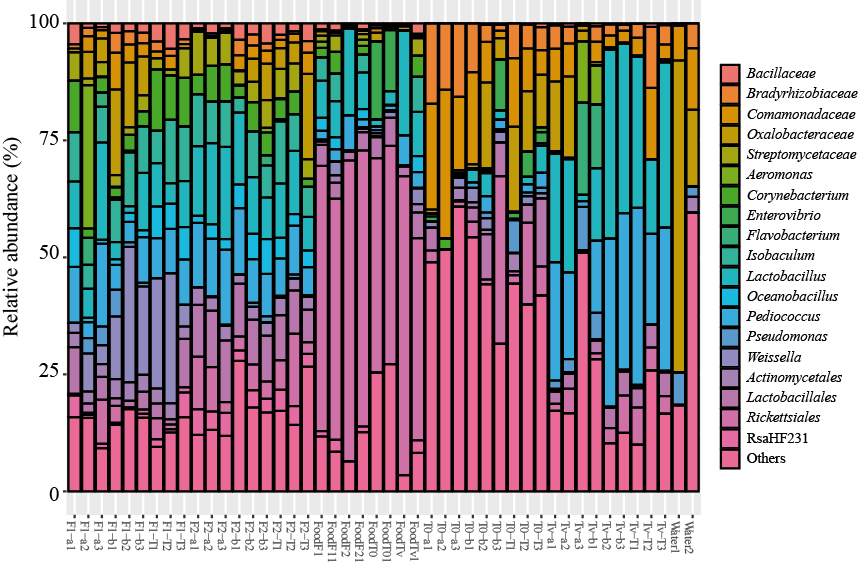


**SUPPLEMENTARY TABLES**

**Supplementary Table S1:** Feed formulations for rainbow trout (g/100 g feed) of commercial-like feed (T_0_), full terrestrial-vegetal feed (Tv) and experimental feeds (F1 and F2).

|  | **Diet designation** | | | |
| --- | --- | --- | --- | --- |
| **Ingredients** | **T_0_** | **TV** | **F1** | **F2** |
| Fish meal | 23.84 |  |  |  |
| Fish oil | 9.00 |  |  |  |
| DHA-rich algae meal |  | 7.57 | 7.48 | 7.46 |
| Yeast meal |  |  | 5.47 | 5.45 |
| Hydrolysed insect meal |  |  | 5.47 | 5.45 |
| Processed animal proteins^a^ | 16.95 |  |  | 5.45 |
| Vegetable oils^b^ |  | 9.00 | 9.00 | 9.00 |
| Plant proteins^c^ | 48.21 | 78.16 | 67.8 | 62.59 |
| Rapeseed lecithin |  | 1.11 | 1.09 | 1.09 |
| Monocalcium phosphate |  | 1.4 | 1.25 | 1.25 |
| Phytase |  | 0.02 | 0.02 | 0.02 |
| Lysine 78% | 0.45 | 1.06 | 0.82 | 0.63 |
| DL Methionine 98% | 0.52 | 0.56 | 0.49 | 0.5 |
| Threonine 98% | 0.23 | 0.22 | 0.22 | 0.22 |
| Vitamin premix | 0.3 | 0.35 | 0.35 | 0.35 |
| Vitamin C Mono P C35 | 0.05 | 0.05 | 0.05 | 0.05 |
| Mineral premix | 0.28 | 0.33 | 0.33 | 0.33 |
| Cholin | 0.17 | 0.17 | 0.16 | 0.16 |
|  | 100 | 100 | 100 | 100 |

^a^ Processed animal proteins: hydrolyzed feather meal, poultry meal, blood meal

^b^ Vegetable oils: rapeseed oil and linseed oil

^c^ Plant proteins: corn gluten, fababean meal, soybean meal, pea protein, guar meal, wheat gluten, soy protein concentrate, rapeseed meal and wheat.

**Supplementary Table S2: Summary of the sequencing pre-processing.** The number and percentage of reads that passed the quality filter, denoising, chimera checking, and the paired-end merged reads are shown.

| **Sample**  **ID** | **Input** | **Filtered** | **Filtered**  **%** | **Denoised** | **Denoised %** | **Merged** | **Merged %** | **Non-chimeric** | **% Non-chimeric** | **% Good reads** |
| --- | --- | --- | --- | --- | --- | --- | --- | --- | --- | --- |
| Buffer-1 | 308894 | 248575 | 80.47 | 248447 | 99.95 | 247927 | 99.79 | 247264 | 99.73 | 80.05 |
| Buffer-2 | 136855 | 97659 | 71.36 | 97544 | 99.88 | 97105 | 99.55 | 94898 | 97.73 | 69.34 |
| F1-a1 | 316696 | 255278 | 80.61 | 254287 | 99.61 | 249230 | 98.01 | 239357 | 96.04 | 75.58 |
| F1-a2 | 401592 | 287741 | 71.65 | 287261 | 99.83 | 285017 | 99.22 | 279752 | 98.15 | 69.66 |
| F1-a3 | 342394 | 246149 | 71.89 | 245295 | 99.65 | 243131 | 99.12 | 239462 | 98.49 | 69.94 |
| F1-b1 | 449646 | 326573 | 72.63 | 326012 | 99.83 | 324314 | 99.48 | 319064 | 98.38 | 70.96 |
| F1-b2 | 330080 | 215163 | 65.19 | 214827 | 99.84 | 213294 | 99.29 | 211016 | 98.93 | 63.93 |
| F1-b3 | 294940 | 211511 | 71.71 | 211174 | 99.84 | 209626 | 99.27 | 205425 | 98 | 69.65 |
| F1-T1 | 331252 | 264445 | 79.83 | 263781 | 99.75 | 262060 | 99.35 | 257434 | 98.23 | 77.72 |
| F1-T2 | 279802 | 227883 | 81.44 | 227490 | 99.83 | 225981 | 99.34 | 222885 | 98.63 | 79.66 |
| F1-T3 | 407324 | 327733 | 80.46 | 326479 | 99.62 | 320596 | 98.20 | 309233 | 96.46 | 75.92 |
| F2-a1 | 326001 | 260963 | 80.05 | 259856 | 99.58 | 254612 | 97.98 | 245453 | 96.40 | 75.29 |
| F2-a2 | 312894 | 252663 | 80.75 | 251069 | 99.37 | 245558 | 97.80 | 234669 | 95.57 | 75 |
| F2-a3 | 370436 | 297053 | 80.19 | 295334 | 99.42 | 288929 | 97.83 | 272366 | 94.27 | 73.53 |
| F2-b1 | 233927 | 187752 | 80.26 | 187515 | 99.87 | 186627 | 99.53 | 184080 | 98.64 | 78.69 |
| F2-b2 | 412998 | 292458 | 70.81 | 291849 | 99.79 | 289930 | 99.34 | 283763 | 97.87 | 68.71 |
| F2-b3 | 202172 | 142837 | 70.65 | 142395 | 99.69 | 141208 | 99.17 | 139452 | 98.76 | 68.98 |
| F2-T1 | 345133 | 247280 | 71.65 | 246721 | 99.77 | 244205 | 98.98 | 239953 | 98.26 | 69.52 |
| F2-T2 | 260150 | 185029 | 71.12 | 184394 | 99.66 | 182672 | 99.07 | 179981 | 98.53 | 69.18 |
| F2-T3 | 423569 | 311289 | 73.49 | 310778 | 99.84 | 308194 | 99.17 | 302944 | 98.30 | 71.52 |
| FoodF1 | 308560 | 215073 | 69.70 | 213964 | 99.48 | 209700 | 98.01 | 181547 | 86.57 | 58.84 |
| FoodF11 | 652999 | 423897 | 64.92 | 421601 | 99.46 | 411383 | 97.58 | 353439 | 85.91 | 54.13 |
| FoodF2 | 327892 | 226189 | 68.98 | 225406 | 99.65 | 221562 | 98.29 | 194052 | 87.58 | 59.18 |
| FoodF21 | 688919 | 477068 | 69.25 | 475086 | 99.58 | 466236 | 98.14 | 399408 | 85.67 | 57.98 |
| FoodT0 | 775140 | 536745 | 69.24 | 535049 | 99.68 | 529555 | 98.97 | 513103 | 96.89 | 66.19 |
| FoodT01 | 882592 | 606761 | 68.75 | 603184 | 99.41 | 591334 | 98.04 | 555710 | 93.98 | 62.96 |
| FoodTv | 346952 | 235411 | 67.85 | 234525 | 99.62 | 230274 | 98.19 | 196860 | 85.49 | 56.74 |
| FoodTv1 | 284469 | 186434 | 65.54 | 185206 | 99.34 | 178163 | 96.20 | 154171 | 86.53 | 54.20 |
| T0-a1 | 188054 | 152947 | 81.33 | 152824 | 99.92 | 152612 | 99.86 | 152424 | 99.88 | 81.05 |
| T0-a2 | 206945 | 168943 | 81.64 | 168849 | 99.94 | 168047 | 99.53 | 167886 | 99.90 | 81.13 |
| T0-a3 | 218078 | 173852 | 79.72 | 173699 | 99.91 | 173532 | 99.90 | 173197 | 99.81 | 79.42 |
| T0-b1 | 237271 | 189615 | 79.91 | 189488 | 99.93 | 188907 | 99.69 | 187518 | 99.26 | 79.03 |
| T0-b2 | 464761 | 337544 | 72.63 | 337206 | 99.90 | 335384 | 99.46 | 334328 | 99.69 | 71.94 |
| T0-b3 | 815773 | 578419 | 70.90 | 577547 | 99.85 | 573137 | 99.24 | 558943 | 97.52 | 68.52 |
| T0-T1 | 266955 | 190299 | 71.29 | 190114 | 99.90 | 188742 | 99.28 | 178557 | 94.60 | 66.89 |
| T0-T2 | 175753 | 125021 | 71.13 | 124738 | 99.77 | 124025 | 99.43 | 122036 | 98.40 | 69.44 |
| T0-T3 | 236300 | 171498 | 72.58 | 171244 | 99.85 | 170649 | 99.65 | 167464 | 98.13 | 70.87 |
| Tv-a1 | 112885 | 77854 | 68.97 | 77562 | 99.62 | 76637 | 98.81 | 76119 | 99.32 | 67.43 |
| Tv-a2 | 64875 | 44890 | 69.19 | 44619 | 99.40 | 43987 | 98.58 | 43859 | 99.71 | 67.61 |
| Tv-a3 | 265855 | 182347 | 68.59 | 181083 | 99.31 | 179233 | 98.98 | 174525 | 97.37 | 65.65 |
| Tv-b1 | 298333 | 239746 | 80.36 | 239466 | 99.88 | 238755 | 99.70 | 236351 | 98.99 | 79.22 |
| Tv-b2 | 281086 | 234385 | 83.39 | 233976 | 99.83 | 231280 | 98.85 | 225906 | 97.68 | 80.37 |
| Tv-b3 | 285706 | 233801 | 81.83 | 233265 | 99.77 | 231830 | 99.38 | 227971 | 98.34 | 79.79 |
| Tv-T1 | 266223 | 210377 | 79.02 | 209965 | 99.80 | 208074 | 99.10 | 204896 | 98.47 | 76.96 |
| Tv-T2 | 183623 | 149692 | 81.52 | 149595 | 99.94 | 149302 | 99.80 | 147738 | 98.95 | 80.46 |
| Tv-T3 | 269735 | 221460 | 82.10 | 221203 | 99.88 | 220016 | 99.46 | 215680 | 98.03 | 79.96 |
| Water1 | 303060 | 255334 | 84.25 | 254746 | 99.77 | 253317 | 99.44 | 247749 | 97.80 | 81.75 |
| Water2 | 453847 | 324395 | 71.48 | 324042 | 99.89 | 321536 | 99.23 | 312215 | 97.10 | 68.79 |
| **Average** | 340612.42 | 251167.31 | 74.63 | 250453.33 | 99.73 | 247654.69 | 98.96 | 237752.15 | 96.52 | 71.24 |

**Supplementary Table S3: PERMANOVA test comparison of bacterial composition under experimental diets.** The distance matrix was based on the Bray-Curtis dissimilarity matrix or Weighted-Unifrac distance. Q-values under 0.05 were considered significant.

|  |  |  |  | **Bray-Curtis** | | | **Weighted-Unifrac** | | |
| --- | --- | --- | --- | --- | --- | --- | --- | --- | --- |
| **Group 1** | **Group 2** | **Sample size** | **Permutations** | **pseudo-F** | **p-value** | **q-value** | **pseudo-F** | **p-value** | **q-value** |
| F1 | F2 | 18 | 999 | 4.254683938 | 0.001 | 0.0012 | 2.6581427 | 0.03 | 0.03 |
| F1 | T_0_ | 18 | 999 | 9.706872833 | 0.001 | 0.0012 | 9.40380102 | 0.001 | 0.0015 |
| F1 | Tv | 18 | 999 | 7.250223727 | 0.001 | 0.0012 | 3.99873549 | 0.002 | 0.0024 |
| F2 | T_0_ | 18 | 999 | 13.66341335 | 0.001 | 0.0012 | 13.4704709 | 0.001 | 0.0015 |
| F2 | Tv | 18 | 999 | 7.773064418 | 0.001 | 0.0012 | 4.58765179 | 0.001 | 0.0015 |
| T_0_ | Tv | 18 | 999 | 8.203779248 | 0.002 | 0.002 | 6.11025901 | 0.001 | 0.0015 |

**Supplementary Table S4:** Crude protein, lipid, fiber, chitin, starch and cinder percentage of each feed formulations for rainbow trout (% of dry matter) of commercial-like feed (T_0_), full terrestrial-vegetal feed (Tv) and experimental feeds (F1 and F2).

|  | **Dry Matter** | **% Proteins** | **% Lipids** | **% Fiber** | **% Chitin** | **% Starch** | **% Cinder** |
| --- | --- | --- | --- | --- | --- | --- | --- |
| T_0_ | 95.90 | 53 | 13 | 2.92 | 0 | 16.3 | 13 |
| T_v_ | 96.38 | 47.51 | 18.11 | 2.93 | 0 | 11.5 | 6.26 |
| F1 | 96.71 | 46.75 | 18.24 | 2.5 | 0.85 | 11.8 | 6.19 |
| F2 | 96.24 | 47.00 | 18.86 | 2.36 | 0.85 | 11.6 | 6.52 |
